# NKCC1 and KCC2: Structural insights into phospho-regulation

**DOI:** 10.3389/fnmol.2022.964488

**Published:** 2022-07-22

**Authors:** Anna-Maria Hartmann, Hans Gerd Nothwang

**Affiliations:** ^1^Division of Neurogenetics, School of Medicine and Health Sciences, Carl von Ossietzky University Oldenburg, Oldenburg, Germany; ^2^Research Center for Neurosensory Sciences, Carl von Ossietzky University Oldenburg, Oldenburg, Germany; ^3^Center of Excellence Hearing4all, Carl von Ossietzky University Oldenburg, Oldenburg, Germany

**Keywords:** CCC, structure, phosphorylation, conformational changes, synaptic inhibition, intrinsically disordered region, neurological diseases

## Abstract

Inhibitory neurotransmission plays a fundamental role in the central nervous system, with about 30–50% of synaptic connections being inhibitory. The action of both inhibitory neurotransmitter, gamma-aminobutyric-acid (GABA) and glycine, mainly relies on the intracellular Cl^–^ concentration in neurons. This is set by the interplay of the cation chloride cotransporters NKCC1 (Na^+^, K^+^, Cl^–^ cotransporter), a main Cl^–^ uptake transporter, and KCC2 (K^+^, Cl^–^ cotransporter), the principle Cl^–^ extruder in neurons. Accordingly, their dysfunction is associated with severe neurological, psychiatric, and neurodegenerative disorders. This has triggered great interest in understanding their regulation, with a strong focus on phosphorylation. Recent structural data by cryogenic electron microscopy provide the unique possibility to gain insight into the action of these phosphorylations. Interestingly, in KCC2, six out of ten (60%) known regulatory phospho-sites reside within a region of 134 amino acid residues (12% of the total residues) between helices α8 and α9 that lacks fixed or ordered three-dimensional structures. It thus represents a so-called intrinsically disordered region. Two further phospho-sites, Tyr^903^ and Thr^906^, are also located in a disordered region between the ß8 strand and the α8 helix. We make the case that especially the disordered region between helices α8 and α9 acts as a platform to integrate different signaling pathways and simultaneously constitute a flexible, highly dynamic linker that can survey a wide variety of distinct conformations. As each conformation can have distinct binding affinities and specificity properties, this enables regulation of [Cl^–^]_*i*_ and thus the ionic driving force in a history-dependent way. This region might thus act as a molecular processor underlying the well described phenomenon of ionic plasticity that has been ascribed to inhibitory neurotransmission. Finally, it might explain the stunning long-range effects of mutations on phospho-sites in KCC2.

## Introduction

**FIGURE 1:**
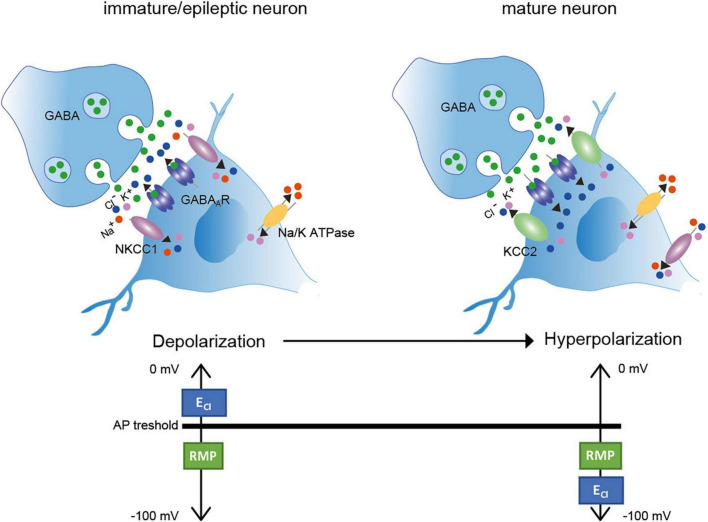
Depolarization/Hyperpolarization shift in inhibitory neurons (Left). In immature neurons, high transport activity of NKCC1 results in increased [Cl^–^]_*i*_. Binding of the inhibitory neurotransmitter GABA to GABA_*A*_ receptors results in Cl^–^ outward currents and thus depolarization. Here, the E_*Cl–*_ is more depolarized than the AP threshold (Right). In mature neurons, KCC2 activity is increased resulting in decreased [Cl^–^]_*i*_. Binding of the inhibitory neurotransmitter GABA to its receptor results in Cl^–^ inward currents and thus hyperpolarization. E_*Cl–*_ is here hyperpolarized according to the resting membrane potential. AP: action potential; RMP: resting membrane potential. Green dots: GABA; red dots: Na^+^; purple dots: K^+^; blue dots: Cl^–^. Figure modified from Moore et al. (2017).

Information transfer in the brain requires a homeostatic control of neuronal firing rate (Turrigiano and Nelson, 2004; Eichler and Meier, 2008). Therefore, a functional balance between excitatory and inhibitory synapses (E-I balance) is established during development and maintained throughout life (Turrigiano and Nelson, 2004; Eichler and Meier, 2008). Excitatory synaptic transmission is mainly mediated through glutamatergic synapses and inhibitory synaptic transmission by GABAergic and glycinergic signaling (Eichler and Meier, 2008). The inhibitory neurotransmitters GABA (gamma aminobutyric acid) and glycine mainly bind to ionotropic GABA_*A*_ and glycine receptors (GABA_*A*_R and GlyR), correspondingly (Bormann et al., 1987). GABA is the main inhibitory neurotransmitter in both the brain and spinal cord, since GABA_*A*_R are widely expressed in these tissues [reviewed in Möhler (2006)]. Glycine is mainly present in the brainstem and spinal cord, where it acts on a variety of neurons involved in motor and sensory function [reviewed in Rahmati et al. (2018)]. In mature neurons, the binding of the inhibitory neurotransmitters results in Cl^–^ influx due to a low intracellular Cl^–^ ([Cl^–^]_*i.*_) concentration and thus to hyperpolarizing inhibitory post-synaptic potentials (Figure 1). In contrast, in immature neurons, binding of GABA and glycine to their respective ionotropic receptors leads to an efflux of Cl^–^ due to a high [Cl^–^]_*i*_ (Cherubini et al., 1990, 1991; Luhmann and Prince, 1991; Zhang et al., 1991; Ehrlich et al., 1999; Ben-Ari et al., 2007; Rahmati et al., 2018; Figure 1). This results in a depolarizing action. The developmental shift from depolarization to hyperpolarization (D/H shift) occurs during early postnatal life (Blaesse et al., 2009; Kaila et al., 2014) and is present throughout the nervous system (e.g., cortex, hippocampus, hypothalamus, brainstem, and spinal cord) (Ben-Ari et al., 1983; Cherubini et al., 1990; Luhmann and Prince, 1991; Wu et al., 1992; Kandler and Friauf, 1995; Owens et al., 1996; Rohrbough and Spitzer, 1996; Ehrlich et al., 1999). However, the timing of the D/H shift can differ between species such as precocial (e.g., guinea pig, prenatal D/H shift) and altricial (e.g., rat and mice, postnatal D/H shift) species (Rivera et al., 1999). Furthermore, even within a species, timing differences exist between different neuronal populations (Löhrke et al., 2005).

Important players to regulate the D/H shift are the secondary active membrane transporters NKCC1 (sodium potassium chloride cotransporter 1) and KCC2 (potassium chloride cotransporter 2) (Delpire, 2000; Payne et al., 2003; Moore et al., 2017; Virtanen et al., 2021). Both transporters mediate the Cl^–^ coupled transport of K^+^ with or without Na^+^ across the plasma membrane. In immature neurons, NKCC1 is one of the main Cl^–^ uptake transporter, maintaining a high [Cl^–^]_*i.*_ (Figure 1; Sung et al., 2000; Ikeda et al., 2004; Dzhala et al., 2005; Achilles et al., 2007). In mature neurons, KCC2 is the essential Cl^–^ extruder that lowers [Cl^–^]_*i*_ and thus enables fast hyperpolarizing post-synaptic inhibition due to Cl^–^ influx (Kaila, 1994; Rivera et al., 1999). NKCC1 is also expressed in mature neurons, but the mRNA expression developmentally changes from a neuronal pattern at birth to a glial pattern (esp. oligodendrocytes and their precursors, endothelial cells, astrocytes and microglia) in adult mouse brain (Hübner et al., 2001a; Su et al., 2001; Wang et al., 2003; Zhang et al., 2014; Henneberger et al., 2020; Virtanen et al., 2020; Tóth et al., 2022). In glia cells, NKCC1 regulates for instance the proliferation and maturation of oligodendrocyte precursor cells in the adult mouse cerebellar white mater (Zonouzi et al., 2015) and modulates the microglial phenotype and inflammatory response (Tóth et al., 2022).

The physiological relevance of NKCC1 and KCC2 is corroborated by the phenotypes present in knock-out mice. Mice with disruption of the gene *Slc12a2* encoding both NKCC1 splice variants (NKCC1a and NKCCb) are viable, but suffer from deafness, pain perception, and male infertility (Randall et al., 1997; Delpire et al., 1999; Delpire and Mount, 2002). Mice with disruption of the gene *Slc12a5* that encodes both splice variants of KCC2 (KCC2a and KCC2b) die shortly after birth due to severe motor deficits that also affect respiration (Hübner et al., 2001b; Uvarov et al., 2007).

Several other plasma membrane Cl^–^ channels and transporters are present to regulate Cl^–^ homeostasis in neurons [see review: (Rahmati et al., 2018)]. These include the voltage-gated Cl^–^ channels (e.g., ClC-1 to 3), Ca^2+^ activated Cl^–^ channels (TMEM16 family, anoctamins), the pH sensitive Cl^–^ channels and transporters of the *SLC4* family [Na^+^- independent Cl^–^/HCO^3–^ exchangers (e.g., AE3) and Na^+^-dependent Cl^–^/HCO^3–^ exchangers (e.g., NCBE and NDCBE)], and *SLC26* family [e.g., anion exchange transporter (*SLC*2*6A7*) and sodium independent sulfate anion transporter (*SLC26A11*)] and glutamate-activated Cl^–^ channels (EAAT4) (Blaesse et al., 2009; Rahmati et al., 2018; Kilb, 2020). In this review, we will focus on the secondary active transporters NKCC1 and KCC2.

## Ionic plasticity

Inhibitory neurotransmission mediated by GABA_*A*_ or glycine receptors is somewhat unique in that its function can be relatively easily modified *via* changes to the ionic driving force. In mature neurons, a low [Cl^–^]_*i*_ results in E_*Cl*_ being slightly hyperpolarized with respect to the neuronal resting membrane potential V_*rest*_ (Figure 1). In P12 auditory neurons of the lateral superior olive, for instance, [Cl^–^]_*i*_ is 8 ± 5 mM, and in cortical pyramidal neurons cultured for 21 days, it is 7.3 ± 0.2 mM (Balakrishnan et al., 2003; Zhu et al., 2005). In such conditions, GABA_*A*_ or glycine receptor activation results in an inward Cl^–^ gradient that reduces excitability by pulling the membrane potential away from threshold. This decreases the probability of action potential generation. However, even relatively small increases in [Cl^–^]_*i*_ will depolarize E_*Cl*_ toward V_*rest*_ (Currin et al., 2020). This significantly reduces or even eliminates hyperpolarizing inhibition thus affecting the input-out function of neurons and modify or even degenerate neuronal function (Currin et al., 2020). Computational models of a mature CA1 pyramidal neuron revealed that shifting the reversal potential of GABA (E_*GABA*_) by only ∼2.5 mM (∼ to 5 mV from −75 to −70 mV) results in an increase in action potential firing by 39% (Saraga et al., 2008). Further increase in Cl^–^can even invert the polarity of GABA_*A*_ or glycine receptor mediated currents from hyperpolarizing to depolarizing. On the other hand, extraordinary decreases in neuronal Cl^–^ with functional relevance have also been observed. Auditory neurons of the superior paraolivary nucleus possess an extremely negative E_*Cl*_, which increases the magnitude of hyperpolarizing currents. This is required to trigger hyperpolarization-activated non-specific cationic and T-type calcium currents to promote rebound spiking to signal when a sound ceases (Kopp-Scheinpflug et al., 2011).

Changes in the ionic driving force for Cl^–^ have been observed on different time scales. The developmental D/H shift occurs on the long term and results in the general observation of hyperpolarizing action of GABA or glycine in the mature brain. More dynamic, short-term alterations have also been reported (Woodin et al., 2003; Khirug et al., 2005; Lamsa et al., 2010; Chamma et al., 2012; Doyon et al., 2016). These changes often occur in a way that relates to the history of synaptic activity. Coincident pre- and post-synaptic spiking results in mature hippocampal neurons in a shift of E_*GABA*_ toward more positive values (Woodin et al., 2003; Ormond and Woodin, 2009). This change in [Cl^–^]_*i*_ in the post-synaptic neurons was synapse specific and dependent on KCC2 activity, as revealed by furosemide application (Woodin et al., 2003). In immature hippocampal neurons, coincident activity was reported to result in both a hyperpolarized E_*GABA*_ (Balena and Woodin, 2008) or a depolarized E_*GABA*_ (Xu et al., 2008). This difference might be attributed to differences in the system used (cultured neurons vs. hippocampal slices) or in the protocols. In both studies, pharmacological approaches related the change in E_*GABA*_ to changes in the activity of NKCC1.

These examples of short-term plasticity that involves changes in the ionic driving force for post-synaptic ionotropic receptors have been referred to as ionic plasticity (Rivera et al., 2005) or ionic shift plasticity (Lamsa et al., 2010). These changes are directly related to the history of activity at inhibitory synapses and likely include rapid post-translational modifications of NKCC1 and KCC2.

## Perturbed [Cl^–^]_*i*_ related diseases

The easy modification of the effect of GABA and glycine *via* changes in the ionic driving force for Cl^–^ makes inhibitory neurotransmission prone to disease causing alterations. Indeed, perturbation of [Cl^–^]_*i*_ is associated with a long and still growing list of neurological, psychiatric, and neurodegenerative disorders including epilepsy, neuropathic pain, spasticity, schizophrenia, autism spectrum disorder, brain trauma, ischemic insults, Rett Syndrome and Parkinson‘s disease (Rivera et al., 2002; Coull et al., 2003; Huberfeld et al., 2007; Papp et al., 2008; Shulga et al., 2008; Boulenguez et al., 2010; Kim et al., 2012; Kahle et al., 2014; Puskarjov et al., 2014; Tyzio et al., 2014; Merner et al., 2015; Ben-Ari, 2017; Pisella et al., 2019; Savardi et al., 2021). These disorders are often associated with increased activity of NKCC1 and/or decreased activity of KCC2 promoting GABA_*A*_R mediated membrane depolarization and excitation (Figure 1; Kaila et al., 2014; Mahadevan and Woodin, 2016; Ben-Ari, 2017; Moore et al., 2017; Fukuda and Watanabe, 2019; Tillman and Zhang, 2019; Liu et al., 2020; Savardi et al., 2021). In patients with temporal lobe epilepsy, a subset of neurons in the subiculum in the hippocampus displayed depolarizing up to excitatory GABAergic response that correlated with decreased KCC2 expression and upregulation of NKCC1 (Cohen et al., 2002; Palma et al., 2006; Huberfeld et al., 2007; Muñoz et al., 2007; Moore et al., 2017). Contradictory, recent finding in NKCC1 knock out mice showed that deletion of NKCC1 results in more severe epileptic phenotype in the intrahippocampal kainate mouse model of temporal lobe epilepsy (Hampel et al., 2021). Thus, NKCC1 role in epilepsy is still not completely understood.

**FIGURE 2:**
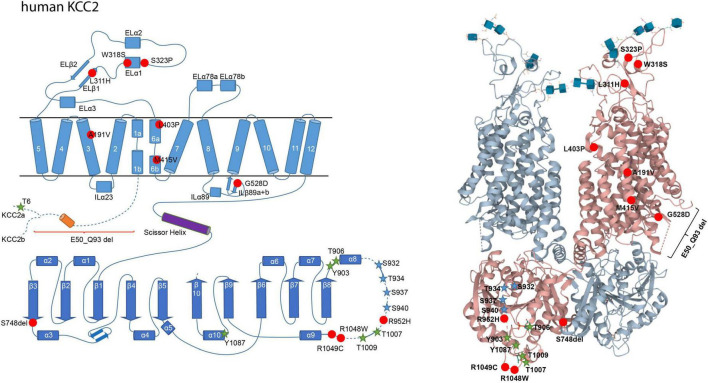
Structural organization of human KCC2. 2-dimensional (left) and 3-dimensional (right) organization of human KCC2 according to Chi X. et al. (2020) (PDB: 6m23). KCC2 consists of 12 transmembrane domains (TMs) and two intracellular termini. A large extracellular loop is located between transmembrane domains 5 and 6 (EL3) and five N-glycosylation sites (blue cubes, left). Phosphorylation sites that increase KCC2 activity upon dephosphorylation are marked as green stars (Thr^6^ in KCC2a, Thr^906^, Tyr^903^, Thr^1007^, Thr^1009^, and Tyr^1087^). Phosphorylation sites that increase KCC2 activity upon phosphorylation are marked as blue stars (Ser^932^, Thr^934^, Ser^937^, Ser^940^). Human pathogenic variants of KCC2 associated with epilepsy, autism-spectrum disorder, and schizophrenia are depicted as red dots (Ala^191^*^Val^*, Leu^311^*^His^*, Trp^318^*^Ser^*, Ser^323^*^Pro^*, Leu^403^*^Pro^*, Met^415^*^Val^*, Gly^528^*^Asp^*, Arg^952^*^His^*, Arg^1048^*^Trp^*, Arg^1049^*^C^*, Ser^748^*^del^*). Annotation of amino acid residues is according to human KCC2b. The 3D reconstruction of KCC2 was generated using cryo-EM (Chi X. et al., 2020). 3D visualization was performed using Mol* Viewer in PDB (Sehnal et al., 2021).

Concerning KCC2, several human pathogenic variants are associated with epilepsy, schizophrenia, and autism spectrum disorder (Figure 2). These include the heterozygous missense mutations of Arg to His at positions 952 (Arg^952His^; numbering according to KCC2b) and 1049 (Arg^1049His^) that are associated with febrile seizures and/or idiopathic generalized seizure and decreased KCC2 activity (Kahle et al., 2014; Puskarjov et al., 2014; Merner et al., 2015). Substitution of Arg^952His^ was also found to be associated with schizophrenia (Merner et al., 2015, 2016). In addition, three autosomal recessive heterozygous mutations (Leu^288His^, Leu^403Pro^, and Gly^528Asp^) were identified in children of two unrelated families, which are associated with epilepsy of infancy with migrating focal seizures (Stödberg et al., 2015). Two children had compound heterozygous mutations of Leu^403Pro^ and Gly^528Asp^ and the other child had a homozygous Leu^288His^ mutation (Stödberg et al., 2015). Leu^403Pro^ and Gly^528Asp^ both result in loss-of-function and Leu^288His^ decreases KCC2 activity (Stödberg et al., 2015). Saitsu et al. (2016) also discovered six heterozygous compound KCC2 variants (E50_Q93*^del^*, Ala^191Val^, Ser^323Pro^, Met^415Val^, Trp^318Ser^, and Ser^748del^) that are associated with this disorder (Saitsu et al., 2016). Analysis of E50_Q93^del^ and Met^415Val^ revealed that each of the mutations strongly decreases KCC2 activity, whereas Ala^191Val^ and Ser^323Pro^ moderately impair KCC2 function. Co-transfection of E50_Q93^del^ with Ala^191Val^ or Met^415Val^ with Ser^323Pro^ significantly decreases KCC2 activity (Saitsu et al., 2016).

**FIGURE 3:**
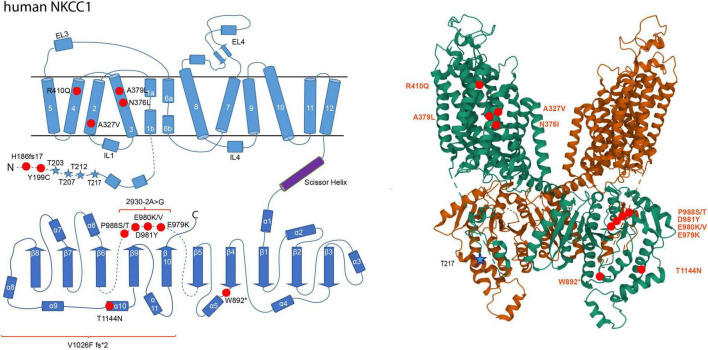
Structural organization of human NKCC1. 2-dimensional (left) and 3-dimensional (right) organization of human NKCC1 (A) according to Zhao et al. (2022) (PDB: 7S1X). NKCC1 consists of 12 transmembrane domains (TMs) and two intracellular termini. A large extracellular loop is located between transmembrane domains 7 and 8 (EL4). Phosphorylation sites that increase NKCC1 activity upon phosphorylation are marked as blue stars (Thr^203^, Thr^207^, Thr^212^, and Thr^217^). Human pathogenic variants of NKCC1 associated with autism spectrum disorder, schizophrenia, multisystem dysfunction, spastic quadriparesis, and hearing impairment are depicted as red dots in human NKCC1 (His^186*fs*17^, Tyr^199^*^Cys^*, Ala^327^*^Val^*, Asn^376^*^Leu^*, Ala^379^*^Leu^*, Arg^410^*^Glu^*, Trp^892*^, Gln^979^*^Lys^*, Asn^981^*^Tyr^*, Pro^988^*^Ser^*, Pro^988^*^Thr^*, Thr^411^*^Asn^*, 2930.2A > G, Val^1026F fs*2^). The 3D reconstruction of NKCC1 was generated using cryo-EM (Zhao et al., 2022). 3D visualization was performed using Mol* Viewer in PDB (Sehnal et al., 2021).

In schizophrenia, an enhanced NKCC1/KCC2 expression ratio was shown to increase [Cl^–^]_*i*_ (Arion and Lewis, 2011; Hyde et al., 2011; Ben-Ari, 2017). Substitution of Arg^952His^ is associated with schizophrenia and results in decreased KCC2 activity (Figure 2; Merner et al., 2015). Additionally, the human pathogenic NKCC1 variant Tyr^199Cys^, which enhances its activity, is associated with this disorder (Figure 3; Merner et al., 2016).

In autism spectrum disorder, downregulation of KCC2 and upregulation of NKCC1 were observed in several brain regions (Savardi et al., 2021). Application of bumetanide, a specific NKCC inhibitor, markedly improves visual contact, sensory behavior, rigidity and memory performance in preclinical trials (Lemonnier and Ben-Ari, 2010; Lemonnier et al., 2012, 2017; Hadjikhani et al., 2015, 2018). This suggests an association of NKCC1 with autism spectrum disorder. This is supported by two human pathogenic variants (Ala^379Leu^ and Arg^410Gln^) that are linked to this disorder and intellectual disabilities (McNeill et al., 2020; Adadey et al., 2021). Both mutations impair NKCC1 function (McNeill et al., 2020), indicating a developmental defect. Unfortunately, bumetanide has a poor blood-brain barrier permeability and two recent phase 3 clinical trials using bumetanide in the treatment of ASD in children and adults showed no effectiveness (Löscher and Kaila, 2021). Concerning KCC2, three human pathogenic variants (Arg^952His^, Arg^1048Trp^, and Arg^1049Cys^) have also been linked to it (Merner et al., 2016). Both Arg^952His^ and Arg^1049Cys^ impair KCC2 function; functional data for Arg^1048Trp^ are not yet available (Kahle et al., 2014).

Several NKCC1 human pathogenic variants are furthermore associated with multisystem dysfunction (Val^1026F^ fs^*2^), spastic quadriparesis (His^186fs17^ frameshift mutant), spastic paraparesis (Asn^376Ile^) and minor developmental delay (W892*) (Delpire et al., 2016; McNeill et al., 2020; Adadey et al., 2021). Finally, NKCC1 exon 21 variants are linked to hearing impairment (Glu^979Lys^, Glu^980Val^, Glu^980Lys^) and hearing loss (Asp^981Tyr^, Pro^988Ser^, Pro^988Thr^, and 2930-2A > G) (Morgan et al., 2020; Mutai et al., 2020; Adadey et al., 2021; Koumangoye et al., 2021; Vanniya et al., 2022). The mutation 2930-2A > G has an effect on splicing leading to loss of exon 21 (Mutai et al., 2020). All of these mutations impair NKCC1 function (Delpire et al., 2016; McNeill et al., 2020; Mutai et al., 2020; Adadey et al., 2021). The human pathogenic variants Ala^327Val^ and Thr^1144Asn^ outside exon 21 are also associated with hearing impairment (McNeill et al., 2020; Adadey et al., 2021). These sensory impairments, however, rather reflects perturbed K^+^ recycling in the inner ear than an imbalance in neurotransmission.

To sum up, dysregulation of NKCC1 and KCC2 result in an imbalance of excitation/inhibition that is associated with several neurological and psychiatric disorders.

## Phospho-regulation of NKCCs and KCCs

Modulation of Cl^–^ extrusion constitute promising new strategies for treating these debilitating diseases. Phosphorylation has emerged as the major means to rapidly and reversibly modulate intrinsic transport activity, cell surface stability, and plasma membrane trafficking of NKCC1 and KCC2 (Kahle et al., 2013). So far, four to five phospho-sites with a regulatory effect on transport activity have been identified in the N-terminus of NKCC1 (Thr^203^, Thr^207^, Thr^212^, and Thr^217^ in human NKCC1; Thr^175^, Thr^179^, Thr^184^, Thr^189^, and Thr^202^ in shark NKCC1) (Muzyamba et al., 1999; Flemmer et al., 2002; Gagnon et al., 2006; Vitari et al., 2006; Hartmann and Nothwang, 2014). For KCC2, the number of regulatory phospho-sites that affect transport activity due to (de)phosphorylation is even higher with one regulatory phospho-site in the N-terminus (Thr^6^ in KCC2a) and nine phospho-sites in the C-terminus (Tyr^903^, Thr^906^, Ser^932^, Thr^934^, Ser^937^, Ser^940^, Thr^1007^, Thr^1009^, and Tyr^1087^) (Lee et al., 2007, 2010; Rinehart et al., 2009; Weber et al., 2014; Titz et al., 2015; Markkanen et al., 2017; Cordshagen et al., 2018; Zhang et al., 2020b). In addition, there are phospho-sites with no detectable effect so far on KCC2 activity (N-terminus: Ser^25^, Ser^26^, Ser^31^, Thr^34^ and C-terminus: Ser^728^, Thr^787^, Thr^999^, Ser^1022^, Ser^1025^, Ser^1026^, Ser^1034^) or which have not yet been functionally investigated (N-terminus: Thr^32^, Ser^55^, Ser^60^, Thr^69^, and C-terminus: Ser^913^, Ser^988^) (Lee et al., 2007; de Los Heros et al., 2014; Weber et al., 2014; Cordshagen et al., 2018; Zhang et al., 2020b). The difference in the location of the phospho-sites between NKCC1 (N-terminus) and KCC2 (C-terminus) might relate to the presence of an autoinhibitory loop present in KCC2 (Chew et al., 2021; Zhang et al., 2021). This loop occludes the translocation pathway and thus locks the transporter in the inactive state (Zhang et al., 2021). The outward-open conformation of the human NKCC1 displays no autoinhibitory loop (Figure 3; Zhao et al., 2022). Although the presence of an auto-inhibitory loop in other conformations cannot be excluded, the current data suggests two distinct regulatory mechanisms in the N-terminus of CCC subfamilies: post-translational modification in NKCC1 and an autoinhibitory loop in KCC2 (Chew et al., 2021).

The high number of regulatory phospho-sites enables the transporters to sample across a multitude of signaling pathways, including with-no-lysine kinase (WNK) with their downstream kinase targets STE20/SP1-related proline/alanine rich kinase (SPAK) and oxidative stress response kinase (OSR1), protein kinase C (PKC), Src-tyrosine kinases, brain type creatine kinases and protein phosphatases (Liedtke et al., 2003; Korkhov et al., 2004; Inoue et al., 2006; Gagnon and Delpire, 2013; de Los Heros et al., 2014; Medina et al., 2014). The high number of phospho-sites might reflect the multi-compartmental organization of a neuron (e.g., soma vs. proximal vs. distal dendrites) and the different states a neuron or a synapse can adopt (see ionic plasticity). Future work should therefore aim to relate individual phospho-sites to specific forms of ionic plasticity. The increasing availability of mice with mutated phospho-sites (Silayeva et al., 2015; Moore et al., 2018, 2019; Pisella et al., 2019) will pave the avenue for such analyses.

## WNK-SPAK/OSR1 mediated phosphorylation of NKCC1 and KCC2

Generally, phosphorylation of NKCC1 and dephosphorylation of KCC2 increase transport activity. The main mechanism that ensures reciprocal regulation is WNK-SPAK/OSR1 dependent phosphorylation of specific NKCC1 and KCC2 phospho-sites, thus activating NKCCs and inactivating KCCs (Darman and Forbush, 2002; Vitari et al., 2006; Richardson et al., 2008; Rinehart et al., 2009; Kahle et al., 2013; Alessi et al., 2014a; Titz et al., 2015; Markkanen et al., 2017; Zhang et al., 2020b). SPAK/OSR1, which is activated *via* WNK1, phosphorylates Thr^6^ and Thr^1007^ of KCC2 (Rinehart et al., 2009; de Los Heros et al., 2014; Conway et al., 2017; Heubl et al., 2017; Markkanen et al., 2017; Moore et al., 2018). WNKs also interact with a yet unknown kinase to phosphorylate Thr^906^ in the KCC2 C-terminus (de Los Heros et al., 2014; Conway et al., 2017). Site directed mutagenesis of Thr^6^ of KCC2a or Thr^906^ and Thr^1007^ of KCC2 to alanine (mimicking the dephosphorylated state) results in activation of KCC2 as shown in cultured hippocampal neurons, cultured cortical neurons and slices, and HEK293 cells (Rinehart et al., 2009; Inoue et al., 2012; Weber et al., 2014; Friedel et al., 2015; Titz et al., 2015). The enhanced activation *via* dephosphorylation of Thr^906^ and Thr^1007^ is accompanied by an increase in cell surface expression in cultured hippocampal neurons (Friedel et al., 2015). Enhanced phosphorylation of Thr^906^ and Thr^1007^ increases in mature hippocampal neurons membrane diffusion resulting in cluster dispersion and enhanced membrane turnover (Heubl et al., 2017; Côme et al., 2019). This indicates that dephosphorylation of these residues increases KCC2 activity. WNK-SPAK/OSR1 mediates also the phosphorylation of human NKCC1 Thr^203^, Thr^207^, Thr^212^, and Thr^217^ resulting in enhanced NKCC1 activity (Darman and Forbush, 2002; Dowd and Forbush, 2003; Moriguchi et al., 2005; Vitari et al., 2006; Gagnon et al., 2007; Richardson and Alessi, 2008; Geng et al., 2009; Thastrup et al., 2012; Alessi et al., 2014b; Hartmann and Nothwang, 2014; Heubl et al., 2017; Shekarabi et al., 2017). Thus, dephosphorylation (KCC2) and phosphorylation (NKCC1) reciprocally decrease the activity of the two Cl^–^ cotransporters (Zhang et al., 2020b).

The reciprocal phosphorylation of NKCC1 and KCC2 by the WNK-SPAK/OSR1-mediated pathway is involved in the regulation of the development-dependent D/H shift. In neurons, WNK1 phosphorylates SPAK at Ser^373^ and of OSR1 at Ser^325^, thereby activating these kinases. This results in phosphorylation of NKCC1 (activation) and KCC2 (inactivation) and thus their reciprocal regulation (Vitari et al., 2005; Richardson and Alessi, 2008; de Los Heros et al., 2014; Moore et al., 2017; Zhang et al., 2020a). The action of WNK1 developmentally decreases, since phosphorylation of Ser^382^ in WNK1, and consequently of its targets Ser^373^ in SPAK and Ser^325^ in OSR1, decreases over time in cortical and hippocampal cultures (Friedel et al., 2015). This causes reduced phosphorylation of Thr^906^ and Thr^1007^ in KCC2 (Rinehart et al., 2009; Friedel et al., 2015; Moore et al., 2017). The developmental dependent dephosphorylation of Thr^906^ and Thr^1007^ activates KCC2 function, shifting E_*GABA*_ to more negative values (Friedel et al., 2015; Moore et al., 2017). This was corroborated by a dominant-negative WNK1 mutant or by genetic depletion of the kinase in immature neurons, as both manipulations cause an early hyperpolarizing action of GABA due to decreased phosphorylation of KCC2 Thr^906^ and Thr^1007^ (Friedel et al., 2015). Moreover, cultured hippocampal neurons derived from a mouse model, in which Thr^906^ and Thr^1007^ were mutated to alanine (mimicking the dephosphorylated state) show an accelerated D/H shift due to increased KCC2 function (Moore et al., 2019). In contrast, Thr^906E^/Thr^1007E^ mice (mimicking phosphorylated states) showed a delayed D/H shift in CA3 pyramidal neurons and hippocampal slices (Pisella et al., 2019). These mice showed in addition long-term abnormalities in social behavior, memory retention and increased seizure susceptibility (Moore et al., 2019; Pisella et al., 2019). These data support the notion that post-translational regulation of KCC2 plays a central role in development-dependent regulation in the D/H shift in the central nervous system and that impairment of this regulatory mechanism entails neurodevelopmental disorders (Pisella et al., 2019).

Reciprocal regulation of NKCC1 and KCC2 is important not only in neuronal development but also in adult neurons. Inhibition of GABA_*A*_R *via* gabazine in mature neurons increases [Cl^–^]_*i*_. This activates WNK1 leading to phosphorylation of Thr^906^/Thr^1007^ in KCC2 (inactivation) and phosphorylation of Thr^203^/Thr^207^/Thr^212^ in NKCC1 (activation) (Heubl et al., 2017). This is important for “auto-tuning” GABAergic signaling *via* rapid regulation of KCC2-mediated Cl^–^ extrusion (Heubl et al., 2017).

## Additional phosphorylation sites in KCC2

The principle that phosphorylation increases the activity of N(K)CCs and *de*phosphorylation that of KCCs is true for N(K)CCs and KCC1, KCC3, and KCC4. Phospho-regulation in KCC2 is more complex since phosphorylation and dephosphorylation can both enhance its activity. Dephosphorylation of the following phospho-sites increases KCC2 activity: Thr^6^ (present only in KCC2a) and Thr^906^, Thr^1007^, Thr^1009^, and Tyr^1087^ (present in both splice variants) (Figure 2). The mechanism leading to phosphorylated Thr^6^, Thr^906^, and Thr^1007^ by WNK1 mediated signaling was already described above. Dephosphorylation of the highly conserved Tyr^1087^ residue increases cell surface stability (Lee et al., 2010) and mutation of Tyr^1087^ to phenylalanine (mimicking the dephosphorylated state) does not alter KCC2 activity (Strange et al., 2000). In contrast, mutation of Tyr^1087^ into aspartate (mimicking the phosphorylated state) abolishes KCC2 activity (Strange et al., 2000; Akerman and Cline, 2006; Watanabe et al., 2009; Pellegrino et al., 2011). This indicates that KCC2 is dephosphorylated at Tyr^1087^ under basal conditions and that phosphorylation of this site decreases KCC2 activity. The highly conserved Thr^1009^ is another site that results in increased activity when dephosphorylated. Mutating this residue into alanine (mimicking the dephosphorylated state) intrinsically increases KCC2 activity without affecting cell surface expression (Cordshagen et al., 2018). The Thr^1009^ phosphorylating kinase has yet to be identified. Thus, several sites have been identified where dephosphorylation increases KCC2 activity.

In contrast, phosphorylation of the following residues activates KCC2: Ser^932^, Thr^934^, Ser^937^, and Ser^940^ (Figure 2). These residues are all encoded by exon 22, which is only present in KCC2 and non-therian KCC4 (Hartmann and Nothwang, 2014). The most in-depth analyzed residue is Ser^940^, which is phosphorylated *via* protein kinase C (PKC) and dephosphorylated *via* protein phosphatase 1 (PP1) (Lee et al., 2007, 2011). Phosphorylation of Ser^940^ increases cell surface expression, transport activity, and membrane clustering of KCC2 (Lee et al., 2007; Chamma et al., 2012), with most clusters found at both excitatory and inhibitory synapses in hippocampal cultures (Chamma et al., 2013; Côme et al., 2019). Accordingly, dephosphorylation of Ser^940^ increases membrane diffusion resulting in cluster dispersion and enhanced membrane turnover of KCC2 (Chamma et al., 2013; Côme et al., 2019). Consequently, its dephosphorylation inactivates KCC2 (Lee et al., 2011). Mutation of Ser^940^ to alanine results in transport activity that is equal or decreased compared to KCC2 wild type activity (Lee et al., 2007; Silayeva et al., 2015; Titz et al., 2015). These different outcomes likely reflect the different cellular systems used for the analyses (HEK293 cells, neuronal cell cultures, or knock-in mice) (Lee et al., 2007; Silayeva et al., 2015; Titz et al., 2015).

During development, phosphorylation of Ser^940^ increases concomitantly with KCC2 activity (Moore et al., 2019). Ser^940Ala^ knock-in mice show a delayed D/H shift, demonstrating that not only dephosphorylation of Thr^906^ and Thr^1007^ is important for the D/H shift, but also phosphorylation of Ser^940^ (Moore et al., 2019). Notably, these mice suffer from profound social interaction abnormalities (Moore et al., 2017, 2019). Furthermore, (de)phosphorylation of Ser^940^ is associated with epilepsy. Induction of status epilepticus using kainate causes dephosphorylation of Ser^940^ and internalization of KCC2 (Silayeva et al., 2015). This observation is supported by an analysis of the two human KCC2 pathogenic variants Arg^952His^ and Arg^1049Cys^. Both variants are associated with idiopathic generalized seizure and decreased Ser^940^ phosphorylation (Kahle et al., 2014; Puskarjov et al., 2014; Silayeva et al., 2015). Phosphorylation of Ser^940^ therefore could provide an approach to limit the progress of status epilepticus (Silayeva et al., 2015).

In addition to Ser^940^, exon 22 encodes the phosphorylation sites Ser^932^, Thr^934^, and Ser^937^. Mutation of any of these residues to aspartate (mimicking the phosphorylated state) intrinsically increases KCC2 activity in HEK293 cells without affecting cell surface expression (Weber et al., 2014; Cordshagen et al., 2018). Mutation into alanine (mimicking the dephosphorylated state) has no effect in HEK293 cells (Weber et al., 2014; Cordshagen et al., 2018). Thus, both dephosphorylation and phosphorylation of specific phospho-sites can increase KCC2 activity. This peculiarity provides KCC2 with a rich regulatory tool-box for graded activity and integration of different signaling pathways (Cordshagen et al., 2018).

## Phosphorylation affects conformation of NKCCs and KCCs

3D structure of the outward-open conformation of human NKCC1 (Figure 3) reveals that the dimeric interface is formed between the C-terminus and the N-terminal phosphoregulatory element and the C-terminus and the TMs (Zhao et al., 2022). These two domains define an allosteric interface that may transmit the impact of (de)phosphorylation of N-terminal phospho-sites, *via* the intervening C-terminal tail and the intracellular loop 1 (ICL1) to affect ion translocation (Zhao et al., 2022). Binding of kinases or phosphatases may form or disrupt these domain interactions (Zhao et al., 2022). However, FRET experiments in NKCC1 revealed that phosphorylation within the N-terminus affects movement of the C-terminus leading to a dissociation of the two monomers within the dimer (Monette and Forbush, 2012). Cross-linking studies support this conclusion. They showed that phosphorylation of residues within the N-terminus affects the localization of TM10 relative to TM12 thereby inducing movement of the C-terminus and disruption of dimerization (Monette et al., 2014; Zhang et al., 2021). Thus, phosphorylation of N-terminal phospho-sites in NKCC1 may induce long-range distance effects involving movement of the C-terminus. It is therefore an open question whether (de)phosphorylation of N-terminal NKCC1 phospho-sites cause disengagement of the TMs as described in the outward-facing cryo-EM of NKCC1 (Zhao et al., 2022) or dissociation of the C-terminal domains (Monette and Forbush, 2012; Monette et al., 2014; Zhang et al., 2021).

(De)phosphorylation dependent conformational differences were also reported for KCC3. To examine the effect of phosphorylation on structural organization, two different KCC3 mutants were generated with triple substitutions of Ser^45^, Thr^940^, and Thr^997^ by either aspartate (KCC3-PM) or by alanine (KCC3-PKO). Analysis by cryo-EM revealed that the “dephosphorylated” KCC3-PKO is more dynamic in the scissor helix region and exhibits a greater rotational flexibility of the C-terminal dimer (Chi G. et al., 2021). The KCC3-PM mutant demonstrated more dynamic conformational changes within the ß7 strand and in the α8 and α10 helices (Chi G. et al., 2021). Multiple conformations for α7 were observed, in which the end of α7 moves 21° outward entailing conformational changes in the α7/ß6 loop (Chi G. et al., 2021). Cryo-EM identified also two conformational states in KCC1, as α8 was observed either above or below α10 (Chi G. et al., 2021). The first state matches the structures of KCC3*^wt^* and KCC3-PM (Chi G. et al., 2021). The second state is stabilized by polar interactions with glutamate residues in α11 (Chi G. et al., 2021). Thus, (de)phosphorylation of C-terminal phospho-sites results in substantial conformational reorganizations within the C-terminus in KCCs.

Notably, KCC2 Thr^906^ and Thr^1007^ correspond to the investigated Thr^940^, and Thr^997^ amino acid residues in KCC3. Both amino acid residues are *bona fide* phospho-sites of KCC2 and targets of the WNK-SPAK/OSR1 signaling pathway with dephosphorylation resulting in increased transport activity (Rinehart et al., 2009; Inoue et al., 2012; de Los Heros et al., 2014; Titz et al., 2015; Markkanen et al., 2017). It is therefore tempting to speculate that changes in their phosphorylation pattern alter the C-terminal conformation of KCC2.

## Intrinsically disordered regions of KCC2 as processors for ionic plasticity

The six KCC2 phosphorylation sites Ser^932^, Thr^934^, Ser^937^, Ser^940^, Thr^1007^, and Thr^1009^, which form a tight cluster, all reside in an intrinsically disordered region (IDR) between α8 and α9 helices according to the cryo-EM reconstruction of KCC2 (Chi G. et al., 2021; Chi X. et al., 2021). The presence of six out of ten (60%) known regulatory KCC2 phospho-sites within a stretch of 134 amino acid residues (12% of the total residues, Met^919^ to Ala^1053^ in *hs*KCC2b) (Figure 2) agrees well with the general enrichment of post-translational modification sites in such regions due to their increased surface area (Oldfield et al., 2008; Forman-Kay and Mittag, 2013; Hsu et al., 2013). In line with this, two further phospho-sites, Tyr^903^ and Thr^906^ are also located in a disordered region between ß8 strand and α8 helix (Figure 2).

**FIGURE 4:**
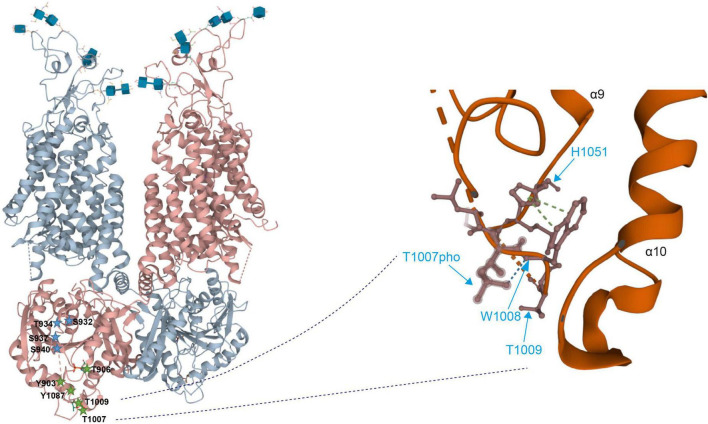
Detailed view on the 3D structure of the KCC2 C-terminus (Left). Overall 3-dimensional organization of human KCC2 (Right). Detailed view of the localization of phosphorylated Thr^1007^ and Thr^1009^. Dashed lines indicate missing density and thus lack of structural information. The 3D reconstruction of KCC2 was generated using cryo-EM (Chi X. et al., 2020). 3D visualization was performed using Mol* Viewer in PDB (Sehnal et al., 2021).

Intrinsically disordered regions do not have a well-defined tertiary structure, instead they are in a dynamic equilibrium between different sets of conformational states (Boehr et al., 2009; Flock et al., 2014). It is thus likely that (de)phosphorylation of the amino acid residues within these regions will induce structural transitions with impact on the conformation of the entire C-terminus (and likely other regions as well). Indeed, phosphorylated Thr^1007^ forms main chain hydrogen bonds with Trp^1008^, that itself has side chain interactions with His^1051^ (pi stacking), and Tyr^903^ forms a main chain hydrogen bond with Ser^899^ (Figure 4). Alterations in phosphorylation might affect these interactions thereby altering the organization and thus conformation of the C-terminus.

**FIGURE 5:**
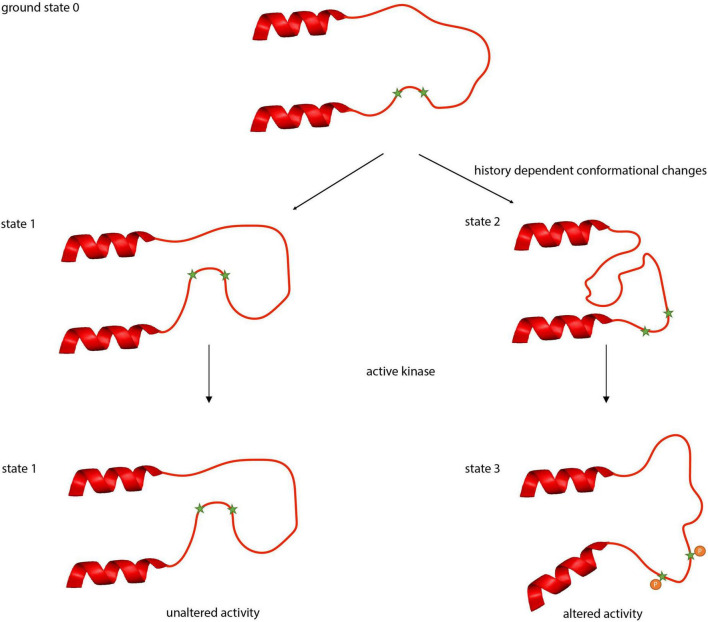
Putative conformational states of the intrinsically disordered regions between α8 and α9 helices in KCC2. Intrinsically disordered regions can adopt a variety of conformational states. Beginning with the ground state 0, different physiological conditions (activity, pH, temperature) can induce different conformational states (states 1 or 2). These conformational changes can result in occlusion (state 1) or deocclusion (state 2) of phospho-sites to signaling pathways. Phosphorylation in the deoccluded state results subsequently in altered transport activity.

The clusters of phospho-sites might not only enable the transporters to integrate multiple signaling pathways but also to regulate activity in a history-dependent manner. Intrinsically disordered regions can adopt a variety of conformations each with distinct binding affinities and specificity properties (Oldfield et al., 2008; Forman-Kay and Mittag, 2013; Hsu et al., 2013; Flock et al., 2014). Thus, starting from a ground state 0, slightly different conformations named states 1 and 2 can be induced by two different physiological states, upon which a signaling pathway will act in different, history-dependent ways. This will induce in one instance a further conformational change resulting in state 3 whereas in the other instance, no further conformational change occurs (Figure 5).

Experiments with the kinase inhibitor staurosporine provide evidence for such different conformational states in KCC2. Mutation of the regulatory phospho-sites Ser^932^ and Thr^1009^ to either alanine or aspartate abrogates stimulation by staurosporine. In contrast, Ser^31^, Thr^34^, and Thr^999^ represent regulatory phospho-sites where only mutation into alanine or aspartate (Ser^31^*^Asp^*, Thr^34^*^Ala^*, and Thr^999Ala^) abrogates stimulation, whereas substitution by the other amino acid residue (Ser^31Ala^, Thr^34Asp^, and Thr^999Asp^) maintains sensitivity to staurosporine (Cordshagen et al., 2018; Zhang et al., 2020b). The change in phosphorylation of either of the three sites likely impacts the accessibility of other phospho-sites such as Ser^932^ and Thr^1009^ to the action of staurosporine (Cordshagen et al., 2018). One conformational state (state 1) might occlude hidden sites that are final targets of the action of staurosporine, resulting in no further activation of KCC2. The other conformational state (state 2) provides access to phospho-sites that are targeted by the action of this reagent, leading to state 3 (Figure 5). This can result in distinct Cl^–^ transport activities, reflecting the past history, and ultimately in different transformations of the neuronal input-output function (Currin et al., 2020), which relate to phenomena as important and diverse as synaptic integration, the flow of information through neuronal circuits, learning and memory, neural circuit development and diseases. The phospho-site enriched unstructured regions are therefore ideally suited to act as a processor to regulate the output of the transporters by computing signaling from ongoing and past physiological states. This inherent feature of an intrinsically disordered region thus might provide a molecular basis for ionic plasticity.

Furthermore, the properties of intrinsically disordered regions might explain the surprising observation of decreased Ser^940^ phosphorylation in the presence of the two human pathogenic variants Arg^952His^ and Arg^1049Cys^ (Kahle et al., 2014; Puskarjov et al., 2014; Silayeva et al., 2015; Figure 2). Both variants may cause altered conformation of the unstructured area, resulting in different binding affinities for PKC and PP1 that determine together the amount of Ser^940^ phosphorylation (Lee et al., 2007, 2011; Kahle et al., 2014). Finally, environmental factors, like changes in temperature, redox-potential and pH can induce conformational changes of intrinsically disordered regions (Kjaergaard et al., 2010; Flock et al., 2014; Jephthah et al., 2019). This might explain the temperature-dependency of KCC2, since increasing the temperature to 37°C decreases KCC2 activity (Hartmann and Nothwang, 2011).

## Conclusion

(De)phosphorylation of phospho-sites most likely results in conformational reorganization as observed for other CCC family members. Many of the phospho-sites in the C-terminus of KCC2 are localized in an unstructured area. Due to biophysical properties of such areas, this part of KCC2 might serve a dual role. It might represent a platform for integrating different signaling pathways and simultaneously constitute a flexible, highly dynamic linker that can survey a wide variety of distinct conformations (Forman-Kay and Mittag, 2013). As each conformation can have distinct binding affinities and specificity properties, this may enable regulation of [Cl^–^]_*i*_ and thus the ionic driving force in a history-dependent way and explain long-range effects of mutations on phospho-sites.

## Author contributions

A-MH and HN equally wrote the manuscript. A-MH generated all of the figures. Both authors contributed to the article and approved the submitted version.

## Conflict of interest

The authors declare that the research was conducted in the absence of any commercial or financial relationships that could be construed as a potential conflict of interest.

## Publisher’s note

All claims expressed in this article are solely those of the authors and do not necessarily represent those of their affiliated organizations, or those of the publisher, the editors and the reviewers. Any product that may be evaluated in this article, or claim that may be made by its manufacturer, is not guaranteed or endorsed by the publisher.

## Abbreviations

CCC: cation chloride cotransporter
KCC: K^+^ Cl^–^cotransporter
NKCC: Na^+^ K^+^ Cl^–^ cotransporter
GABA: gamma-aminobutyric acid
TM: transmembrane domain.
